# Predicting optimal treatment allocation for cognitive analytic‐guided self‐help versus cognitive behavioural‐guided self‐help

**DOI:** 10.1111/bjc.12508

**Published:** 2024-10-23

**Authors:** Caroline Wojnarowski, Melanie Simmonds‐Buckley, Stephen Kellett

**Affiliations:** ^1^ University of Sheffield Sheffield UK; ^2^ Swallownest Court Rotherham, Doncaster and South Humber NHS Foundation Trust Doncaster UK

**Keywords:** anxiety, cognitive analytic therapy, cognitive behavioural therapy, guided self‐help, machine learning, patient advantage index, patient preference

## Abstract

**Objectives:**

Given the ubiquity in routine services of low‐intensity guided self‐help (GSH) psychological interventions, better patient selection for these brief interventions would be organizationally efficient. This study therefore sought to define who would respond best to two different types of GSH for anxiety to enable better future treatment matching.

**Methods:**

The study used outcome data from a patient preference trial (*N* = 209) comparing cognitive analytic therapy‐guided self‐help (CAT‐GSH) with cognitive behavioural therapy‐guided self‐help (CBT‐GSH). Elastic Net regularization and Boruta random forest variable selection methods were applied. Regression models calculated the patient advantage index (PAI) to designate which GSH was likely the most effective for each patient. Outcomes were compared for those receiving their PAI‐indicated optimal and non‐optimal GSH.

**Results:**

Lower baseline depression and anxiety severity predicted better outcomes for both types of GSH. Patient preference status was not associated with outcome during either GSH. Sixty‐three % received their model indicating optimal GSH and these had significantly higher rates of reliable and clinically significant reductions in anxiety at both post‐treatment (35.9% vs. 16.6%) and follow‐up (36.6% vs. 19.2%). No single patient with a large PAI had a reliable and clinically significant reduction in anxiety at post‐treatment or follow‐up when they did not receive their optimal GSH.

**Conclusions:**

Treatment matching algorithms have the potential to support evidenced‐based treatment selection for GSH. Treatment selection and supporting patient choice needs to be integrated. Future research needs to investigate the use of the PAI for GSH treatment matching, but with larger and more balanced samples.

**Practitioner points**

- Matching patients to their optimal GSH results in better outcomes particularly when the PAI‐predicted benefit is large.
- Patient choice should still be a factor in the treatment plan, particularly when an optimal treatment is not empirically identified for the patient.
- Future research is needed with larger, more balanced samples, to develop an algorithm to predict optimal treatments for GSH delivered in NHS Talking Therapies services.

## INTRODUCTION

The National Institute of Clinical Excellence (NICE) recommends cognitive behavioural therapy (CBT) for the treatment of anxiety (NICE, 2011). To meet increased demand for talking therapies, CBT has been adapted into brief and low‐intensity, guided self‐help (GSH) formats (Bennett‐Levy et al., 2010). The Improving Access to Psychological Therapies (IAPT – now National Health Service [NHS] Talking Therapies) uses stepped‐care, so that CBT‐GSH is first delivered to patients with mild–moderate anxiety. CBT‐GSH is provided by psychological well‐being practitioners (PWPs) over 6–8 (35‐min) sessions, and their clinical role is that of being a psychoeducational coach (Turpin, 2010). GSH is supported rather than pure self‐help, because the support aspect consistently produces better results (Lewis et al., 2012).

There is a variable evidence base concerning the efficacy of CBT‐GSH for anxiety. The Cuijpers et al. (2010) meta‐analysis found CBT‐GSH to be comparable to CBT and this was mirrored by Priemer and Talbot (2013). However, the Coull and Morris (2011) meta‐analysis found that while CBT‐GSH was effective at post‐treatment, this effect became attenuated at follow‐up. High dropout rates (Chan & Adams, 2014) suggest poor treatment acceptability (Milosevic et al., 2015) and there is evidence of high relapse rates following GSH (Delgadillo et al., 2018). This evidence highlights the need for patients to be offered at step 2 of Talking Therapies services rapid access to a wider choice of evidenced‐based and acceptable GSH, that then have a durable clinical impact.

Therefore, Meadows and Kellett (2017) developed a manualized version of cognitive analytic therapy‐guided self‐help (CAT‐GSH). The evidence base for CAT for anxiety is supported by clinical trials (Boogar et al., 2013) and cohort studies (Tzouramanis et al., 2010). CAT‐GSH has high adherence to GSH principles, is based on the three‐phase CAT structure (i.e., reformulation, recognition and revision), generates low dropout rates, is easy for PWPS to deliver and is clinically effective with a durable short‐term effect (Meadows & Kellett, 2017; Wray et al., 2022). When compared to CBT‐GSH in a patient preference clinical trial, CAT‐GSH was equally efficacious, was more acceptable and also generated model‐specific types of idiographic change (Headley et al., 2024; Kellett et al., 2023). However, there was variability in recovery rates in the trial (i.e., 42.4% of CAT‐GSH patients met reliable recovery compared to 50.9% of CBT‐GSH patients) and this raises the question of whether these rates could have been improved by better treatment matching?

Precision mental health care employs data‐driven methods to monitor patients' treatment response, models the predicted intervention prognosis and so aims to personalize treatment for individual patients (Delgadillo & Lutz, 2020). Prediction models have been integrated into routine practice across medicine and regularly support clinical guidelines around treatment allocation in these settings (Damen et al., 2016; NICE, 2014). In terms of mental health, there are examples of prediction algorithms being used to identify who would have the best treatment response to CBT or psychodynamic therapy (Schwartz et al., 2021), CBT or eye movement desensitization and reprocessing (Deisenhofer et al., 2018) and CBT or interpersonal psychotherapy (Huibers et al., 2015). It is worth noting that such studies were on traditional psychotherapies and in trials where participants are allocated to treatment via randomization (i.e., there was no patient choice). Machine learning methods are becoming increasingly popular to enhance variable selection within prediction models, with increasing generalizability to new samples (Delgadillo et al., 2017).

DeRubeis et al. (2014) developed an approach, the *personalized advantage index* (PAI), that integrates multiple identified outcome predictors from different treatments into one statistical model. The PAI identifies in two or more comparable treatments, the intervention that is more effective for an individual by producing counterfactual outcome predictions. The intervention that an individual is predicted to respond to better is then considered their ‘optimal treatment’ and this also enables outcomes from optimal and non‐optimal treatments subsequently delivered to be compared. For example, the PAI in DeRubeis et al. (2014) predicted a clinically meaningful advantage for 60% of patients assigned to their predicted optimal treatment. Headley et al. (2024) recently called for evidenced‐based methods such as the PAI to improve allocation of patients in routine practice to differing versions of efficacious GSH.

The patient preference trial of CBT‐GSH versus CAT‐GSH for anxiety delivered at step‐2 NHS Talking Therapies collected routine NHS Talking Therapies outcome data alongside the primary outcomes of the trial (Kellett et al., 2023). The data from this clinical trial have been used in the current study to develop predictive models of treatment outcome. To summarize, Kellett et al. (2023) found that were no significant differences in outcome at post‐treatment and 24‐week follow‐up on the Beck Anxiety Inventory (BAI; Beck & Steer, 1993) between CAT‐GSH and CBT‐GSH. This suggests that CAT‐GSH is comparable in efficacy to CBT‐GSH as the ‘treatment as usual’ at step 2. The main objective of the current study was to develop and test a personalized treatment selection method to match patients to their optimal GSH. This therefore is the first test of the PAI in which it is being used to assess whether an algorithm can better select patients for GSH, particularly when they have specifically chosen the GSH they receive. The study had three key aims: (1) to use a variable selection procedure to identify baseline characteristics that significantly predict treatment outcome for CBT‐GSH and CAT‐GSH, (2) to use identified predictors to develop separate predictive models for each GSH and calculate the PAI to indicate the optimal GSH for each patient and (3) to assess the efficacy of the models by comparing treatment response for patients who then went onto receive their optimal versus non‐optimal GSH. It was hypothesized that patients who received their optimal GSH as indicated by the PAI would have better outcomes than patients who received their non‐optimal GSH.

## METHOD

### Ethics and setting

Ethical approval was achieved for the secondary data analysis (ref: 050758). The study is reported according to the Transparent Reporting of Multivariable Prediction Model for Individual Prognosis or Diagnosis (TRIPOD + AI) guidelines (Collins et al., 2015). The clinical trial conducted by Kellett et al. (2023) was a partially randomized patient preference trial (Torgerson & Sibbald, 1998) sited in a routine NHS IAPT service and the trial protocol was published (Kellett, Bee, et al., 2021). When deemed appropriate for a step 2 intervention in the service during a telephone triage, patients were given the opportunity to participate in the trial. If they accepted, a trial eligibility interview was completed where informed written consent was taken. If patients met eligibility criteria (see Appendix S1), they were offered randomization, or they could choose CBT‐GSH or CAT‐GSH by preference after reading an information sheet describing the approach taken in each GSH.

### Interventions

GSH was delivered by qualified and accredited PWPs over the telephone due to the COVID‐19 pandemic. All PWPs had passed an NHS Talking Therapies 1‐year post‐graduate certificate in CBT‐GSH following a national curriculum (UCL, 2014) and attended a 2‐day CAT‐GSH training session as part of the study. PWPs had 1‐h per week of individual case management supervision and were enrolled in group supervision monthly for 2 h for each type of GSH during the study. Both interventions had a contract of 6–8 sessions, which were each 35‐min long. CBT‐GSH is a low‐intensity, structured psychological intervention based on the principles of CBT and works in the ‘here and now’. Treatment followed the NHS Talking Therapies CBT‐GSH treatment protocol (Richards & Whyte, 2011) and this is treatment as usual within step 2 of NHS Talking Therapies. The patient works through standardized worksheets (NICE, 2009, 2011) during CBT‐GSH focusing on understanding and then changing current anxious thoughts, feelings and behaviours. CAT‐GSH is a low‐intensity, structured psychological intervention workbook based on CAT principles and has a ‘past‐present’ focus (Meadows & Kellett, 2017). Both versions of GSH therefore had a psychoeducational workbook, but these differed in terms of content, style and focus. CBT‐GSH requires an effective therapeutic relationship but does not use transference/countertransference, whereas CAT‐GSH works within the therapeutic relationship and makes use of transference/countertransference (Meadows & Kellett, 2017). As part of the trial, the GSH sessions in each arm were assessed using a validated measure of GSH (Kellett, Bee, et al., 2021; Kellett, Simmonds‐Buckley, et al., 2021) and both forms of GSH were being delivered in a competent manner and there were no differences between CBT‐GSH and CAT‐GSH in terms of competency levels.

### Data collection

In total, *N* = 271 patients were eligible for inclusion and were allocated to either CBT‐GSH or CAT‐GSH. Patients completed trial measures at baseline (week 0), post‐treatment (week 8) and follow‐up (week 24). Four clinical outcome measures were completed – Beck Anxiety Inventory (BAI; Beck & Steer, 1993); Generalized Anxiety Disorder‐7 (GAD‐7; Kroenke et al., 2007); Patient Health Questionnaire‐9 (PHQ‐9; Kroenke et al., 2001); Work and Social Adjustment Scale (WSAS; Mundt et al., 2002). See Appendix S2 for the data collected within the initial trial and suitable for current analysis. See Appendix S3 for a description of the measures. The main trial used the BAI as the primary outcome measure. This current study used the GAD‐7 as the primary outcome measure as it is part of the IAPT minimum dataset, therefore maximizing potential generalizability.

### Sample characteristics and sample size

The definition of a treatment episode in NHS Talking Therapies (National Collaborating Centre for Mental Health, 2018) is ≥2 sessions, so this was the criterion used here. This created a sample of *N* = 209 (i.e., 62 cases excluded). More patients accessed CAT‐GSH (*n* = 154) than CBT‐GSH (*n* = 55); 93.8% of patients were allocated to GSH via preference rather than randomization. There was no difference between the two versions of GSH on number of sessions attended (*t*(207) = 2.93, *p* = .642), despite CAT‐GSH (*M* = 5.97, SD = 2) participants attending slighter more sessions (*M* = 5.04, SD = .06). Appendix S4 contains the STROBE summary chart (Von Elm et al., 2017) of sample selection. Table 1 presents a summary of sample characteristics and comparisons between CAT‐GSH and CBT‐GSH. The samples were mostly matched, apart from significantly more women receiving CAT‐GSH. There was also a significant difference in terms of previous treatment, suggesting that more patients preferring CAT‐GSH had received a previous treatment.

**TABLE 1:** Summary of sample characteristics.

	Full sample (*N* = 209)	CBT‐GSH (*N* = 55)	CAT‐GSH (*N* = 154)	Test statistic	*p*
Demographics					
Females^2^	75.6%	65.5%	79.2%	*X* ^*2*^(1) = 4.16	.041
Age^1^	36.49 (13.81)	36.18 (13.97)	36.60 (13.80)	*t* (207) = .19	.846
Ethnicity^2^					
White British	90.4%	94.5%	89%	*X* ^*2*^(1) = 1.46	.227
Other	9.6%	5.5%	11%		
IMD decile^1^					
1 = Poorest 10 = Affluent	4.14 (2.77)	4.05 (2.82)	4.17 (2.76)	*t* (207) = .26	.793
Unemployed^2^	12.9%	12.7%	13%	*X* ^*2*^(1) = .002	.961
Perinatal	6.2%	5.8%	7.3%	*t* (207) = .38	.354
Heterosexual	90%	89.6%	90.9%	*t* (207) = .27	.392
Previous CAT	1.4%	0	1.9%	*t* (207) = 1.04	.150
Allocation Choice^2^					
Preference	93.8%	90.9%	94.8%	*X* ^*2*^(1) = 1.05	.304
Randomized	6.2%	9.1%	5.2%		
Baseline severity measures					
GAD‐7^1^	13.62 (4.81)	14.24 (4.67)	13.40 (4.85)	*t* (207) = −1.11	.267
PHQ‐9^1^	13.65 (5.59)	14.09 (4.68)	13.49 (5.89)	*t* (207) = −.68	.498
WSAS^1^	18.69 (8.57)	17.89 (9.23)	18.97 (8.33)	*t* (207) = .80	.422
BAI^1^	25.50 (9.82)	25.69 (9.57)	25.43 (9.94)	*t* (207) = −.17	.865
LTC^2^	31.6%	34.5%	30.5%	*X* ^*2*^(1) = .30	.581
Previous Treatment^2^	45.9%	25.5%	53.2%	*X* ^*2*^(1) = 12.61	<.001
Medication^2^	56.5%	61.8%	54.5%	*X* ^*2*^(1) = .87	.350

*Note*: 1 = Mean and Standard Deviation; 2 = Percentages.

Abbreviations: BAI, Beck's Anxiety Inventory; CAT‐GSH, guided self‐help cognitive‐analytic therapy; CBT‐GSH, guided self‐help cognitive‐behavioural therapy; GAD‐7, Generalized Anxiety Disorder Questionnaire; IMD Decile, index of multiple deprivation in deciles; LTC, long‐term condition; PHQ‐9, Patient Health Questionnaire; WSAS, Work and Social Adjustment Scale.

The study sample was *N* = 209, but this was unequal (i.e., 55 CBT‐GSH vs. 154 CAT‐GSH). The CBT‐GSH group fell short of the sample size calculation for suitable power (see Appendix S5) and so keeping a subset of the data for an external cross‐validation was not feasible. Using the full dataset maximized power for development of the models (DeRubeis et al., 2014).

### Data preparation

The R package ‘missForest’ (Stekhoven & Bühlmann, 2012) in R (R Core Team, 2021) and Rstudio (Rstudio Team, 2021) was used to input missing data using a random forest approach separately for both GSH formats. Marital status was removed as a candidate predictor as it had too much missing data to be reliably imputed (86%). Categorical variables were collapsed into binary variables (e.g., employment status became unemployed or employed/other; sexual orientation became heterosexual or not heterosexual). Continuous variables were standardized into Z‐scores and binary variables were also dummy coded as −.5 and .5.

### Variable selection

Data were separated into CAT‐GSH and CBT‐GSH subsets for the purpose of variable selection to build predictive models. Two machine learning approaches (i.e., a decision tree method and a penalized regression method) selected variables in each intervention dataset. Regression predictions were compared using evaluation metrics to identify the best fitting model. A total of 18 variables were included as predictors in each model, with post‐treatment GAD‐7 scores as the dependent variable. Baseline GAD‐7 scores were not included in this analysis as it was later included in the regression models by forced entry to control for anxiety levels at screening (Moggia et al., 2023).

The Boruta approach handles multivariate interactions and was conducted in R using the ‘Boruta’ package (Kursa & Rudnicki, 2010). Boruta is a form of random forest, which is a supervised machine learning algorithm that builds multiple decision trees using a bagging method (i.e., a combination of variable and bootstrapping samples; Breiman, 2001). This includes shadow variables (e.g., one continuous, one categorical) based on the distributions of other variables in the dataset and included in the model as a ‘noise’ variable (i.e., have no actual predictive power). Only predictor variables which are ranked higher than one (tentative inclusion) or both (confirmed inclusion) shadow variables are deemed to have reliable predictive power over and above noise and are therefore retained (see Appendix S6). There are known issues of biased importance values and overfitting when using random forest approaches, particularly when there are a smaller number of candidate variables (Tang et al., 2018). Therefore, a second variable selection method was also used: elastic net regularization variable selection (Zou & Hastie, 2005). Elastic net is a linear regression technique that uses a penalty term to shrink coefficients of predictors that are unimportant. It identifies variables that are reliably associated with an outcome, but also adds ‘weight’ to variables with stronger or weaker predictive value.

Variables identified by each model were entered into separate linear regressions to produce predicted outcomes. Leave‐one‐out cross‐validation (LOOCV) was used to prevent over‐fitting (Efron, 1982). LOOCV estimates each model without information about the participant whose score is being predicted, therefore uses a sample of (*n*−1), with *n* being the sample size. This aims to reduce bias in predicted values. Analysis was performed using the ‘caret’ package (Kuhn, 2008) within R. Predicted outcomes from each regression model were compared with the observed outcomes and evaluation metrics were compared. The model with the lowest error and highest correlation between actual and predicted scores was chosen as the preferred model.

### PAI estimation

The preferred regression model for each GSH was used to predict post‐treatment GAD‐7 scores for both CAT‐GSH and CBT‐GSH in the full sample. This produced a predicted score for both treatment modalities for each patient. The PAI was estimated for every individual patient by calculating the difference between their predicted post‐treatment GAD‐7 score for each treatment (i.e., positive PAI value indicated greater benefit from CAT‐GSH; a negative PAI indicated greater benefit from CBT‐GSH). Patients who had received the GSH intervention recommended by the PAI were classified into the optimal treatment group (based on +/−), whereas those who did not, were classified into the non‐optimal group (Moggia et al., 2023). Observed post‐treatment and follow‐up GAD‐7 scores and reliable and clinically significant improvement (RCSI) rates were compared between the optimal and non‐optimal groups using mixed analysis of variance (ANOVA; for baseline, post‐treatment and 24‐week follow‐up timepoints) and Chi‐squared tests (post‐treatment and 24‐week follow‐up) respectively. RCSI was defined as GAD‐7 change score ≥4 and the post‐treatment/follow‐up score being below the clinical cut‐off of 8 (Jacobson et al., 1999; National Collaborating Centre for Mental Health, 2018). Data were analysed to assess assumptions required for an ANOVA using the Shapiro‐Wilks and Levene's Tests (see Appendix S7). Some of the data were not normally distributed, but due to the robustness of ANOVA, the analysis was retained (Blanca et al., 2017). To explore the impact on outcomes in those who had the biggest indicated PAI benefit, a between‐subgroup secondary analysis was conducted based on optimal and non‐optimal groups that consisted of patients with a PAI value greater than one standard deviation (SD) larger than the sample mean (Delgadillo & Lutz, 2020). If the PAI was smaller than this sum, no optimal treatment was indicated.

## RESULTS

### Variable selection and estimation of LOOCV regressions

Following Boruta variable selection, five variables (27.78%) were selected as potential predictors (i.e., previous CBT, sexual orientation, baseline BAI score, baseline PHQ‐9 and baseline WSAS) for CAT‐GSH. Four variables (22.22%) were selected as potential predictors (i.e., employment status, baseline BAI, indices of multiple deprivation and baseline PHQ‐9) for CBT‐GSH. Following the elastic net variable selection, eight variables (44.44%) were selected as potential predictors (i.e., baseline PHQ‐9, baseline WSAS, baseline BAI, long‐term condition, perinatal status, sexual orientation, previous CAT and previous CBT) for CAT‐GSH. Five variables (27.78%) were selected as potential predictors (i.e., indices of multiple deprivation, baseline PHQ‐9, baseline BAI, ethnicity and employment status) for CBT‐GSH. Table 2 contains the evaluation metrics for the LOOCV regressions for each variable selection model. The elastic net models outperformed Boruta on all metrics for CAT‐GSH. Due to both methods selecting the same variables for CBT‐GSH, all evaluation metrics were identical. Therefore, the elastic net variable selection was selected as the preferred model for both formats of GSH to ensure congruence.

**TABLE 2:** LOOCV regression results.

	RMSE	*R* ^2^	MAE	*r*
CAT‐GSH				
Boruta	4.09	.32	3.21	.62
Elastic Net	4.03	.33	3.12	.64
CBT‐GSH				
Boruta	4.50	.10	3.62	.54
Elastic Net	4.50	.10	3.62	.54

### Final selected models

Prognostic variables across the two versions of GSH were baseline PHQ‐9 scores and BAI scores with lower scores suggesting better outcomes. For CBT‐GSH, patients from a higher socioeconomic status were more likely to have better outcomes, along with being White British and unemployed. For CAT‐GSH, better treatment outcomes were associated with lower baseline WSAS scores, a self‐reported long‐term condition, the perinatal period and identifying as not heterosexual. Previously engaging in CBT was associated with poorer outcomes, whilst previously engaging in CAT was associated with better outcomes.

### Optimal guided self‐help

**FIGURE 1:**
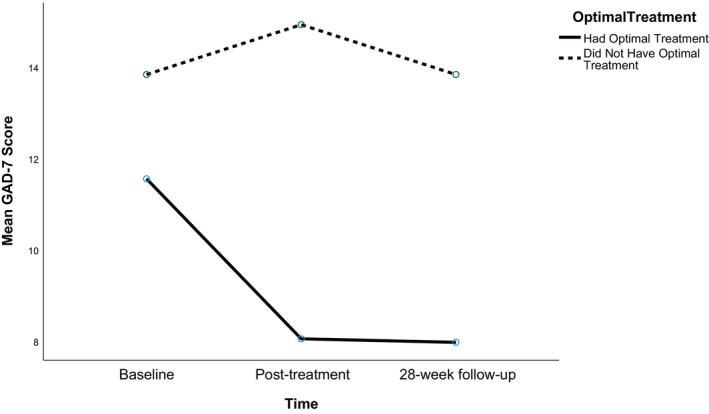
Mean GAD‐7 scores across timepoints for the optimal versus non‐optimal GSH groups in the full sample.

Based on +/− PAI values in the full sample (*N* = 209), CBT‐GSH was indicated as the optimal treatment for 34.9% and CAT‐GSH for 65.1%. Therefore, 62.7% received their optimal GSH and 37.3% did not. There was a significant between subjects' effect of having received optimal GSH on GAD‐7 outcomes (*F*(1, 207) = 21.675, *p* < .001); the partial Eta squared (*η* ^2^ = .10) suggested a medium effect size (Miles & Shelvin, 2001). Mauchly's test indicated that the assumption of sphericity had been violated within the repeated measures analysis (*χ* ^2^ (2) = 39.62, *p* = <.001). Thus, the Greenhouse–Geisser corrected results were reported. There was a significant within subjects' effect of time on GAD‐7 outcomes [*ε* = .85, *F*(1.7, 352.35) = 117.54, *p* < .001, *η* ^2^ = .36] suggesting a large effect size. However, the interaction effect between GAD‐7 scores over time and receiving optimal GSH was non‐significant [*ε* = .85, *F*(1.7, 352.35) = 1.29, *p* = .273]. Figure 1 displays anxiety scores at baseline, post‐treatment and follow‐up for patients receiving optimal versus non‐optimal GSH.

At post‐treatment, significantly more patients who had their optimal GSH (35.9%) met RCSI compared to patients (16.6%) who did not (*X* ^2^ (1, *N* = 209) = 8.82, *p* = .003). The odds‐ratio indicated that patients who had their PAI recommended GSH were more than twice as likely to recover (OR = .36). The same pattern was observed at 24‐week follow‐up, with significantly more patients (*X* ^2^ (1, *N* = 209) = 7.04, *p* = .008) who had their optimal GSH (36.6%) experiencing RCSI compared (19.2%) to those who did not. This indicates that receiving the PAI recommended GSH more than doubled the chance of longer term recovery (OR = .41).

### Subgroup analysis

**FIGURE 2:**
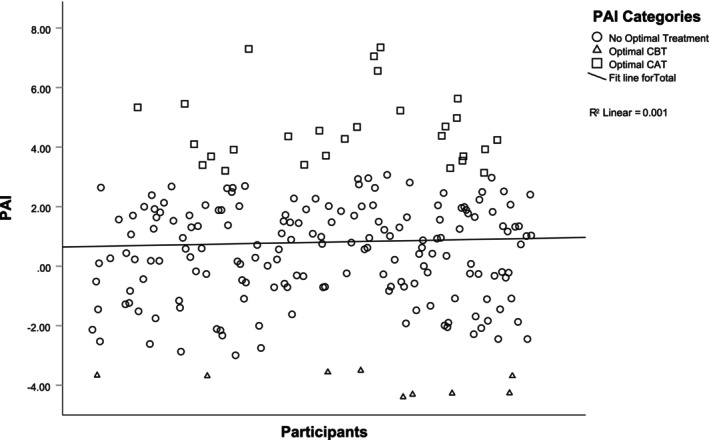
Distribution of optimal GSH.

**FIGURE 3:**
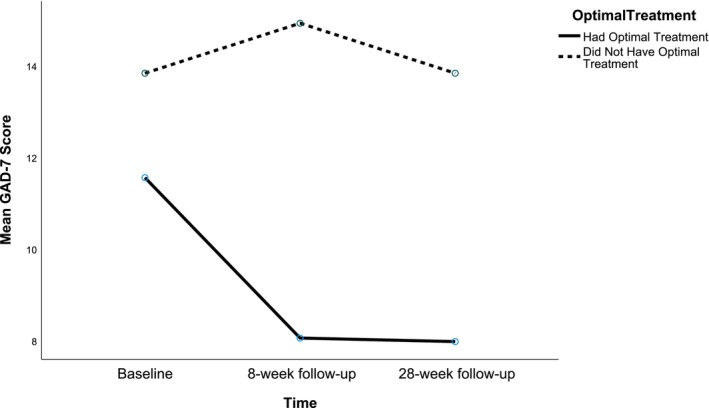
Mean GAD‐7 scores across timepoints for the optimal versus non‐optimal GSH groups in the subgroup.

A subgroup (*N* = 37) who had the largest indicated PAI benefit (PAI ≥ 3.08 [mean ± 1 SD]) was identified. CBT‐GSH was indicated as the optimal treatment for 4.3%, CAT‐GSH indicated as the optimal treatment for 13.4% and no optimal treatment was identified for 82.3%. Classifications indicated 70.3% received their optimal GSH and 29.7% did not received their optimal GSH. Figure 2 displays distribution of the PAI for this subgroup. There was a significant between subjects' effect of receiving optimal GSH on GAD‐7 outcomes (*F*(1, 35) = 12.296, *p* = .001); the partial Eta squared (*η* ^2^ = .26) suggested a large effect size (Miles & Shelvin, 2001). Mauchly's test indicated that the assumption of sphericity had been violated (*χ* ^2^ (2) = 14.94, *p* = <.001). Thus, the Greenhouse–Geisser corrected results were again reported. There was a non‐significant within subjects' effect of time on GAD‐7 outcomes (*ε* = .78, *F*(1.48, 51.64) = 2.19, *p* = .119). The interaction effect between GAD‐7 scores over time and receiving optimal GSH, was significant (*ε* = .78, *F*(1.48, 51.64) = 3.83, *p* = .040, *η* ^2^ = .01), suggesting a medium effect size. Figure 3 displays the mean GAD‐7 scores at baseline, post‐treatment and follow‐up for patients who received optimal versus non‐optimal GSH.

For those patients that did not have their optimal GSH, none met RCSI criteria at post‐treatment or 24‐week follow‐up. For patients that had their optimal GSH, then 30.8% met RCSI and this difference was significant (*X* ^2^(1, *N* = 37) = 4.32, *p* = .038). The odds ratio also suggests that patients who had their optimal GSH had a better outcome (OR = .69). At post‐treatment, significantly more of these patients (*X* ^2^(1, *N* = 37) = 4.32, *p* = .038) who received their optimal GSH met RCSI (30.8%) compared to patients who did not (0%). The odds ratio indicated that patients who received their PAI recommended GSH were more likely to recover (OR = .69).

### Patient preference

For those patients that the PAI recommended CAT‐GSH, 74.26% stated a preference for CAT‐GSH. Of those recommended CBT‐GSH by the PAI, 61.64% preferred CAT‐GSH. This suggests that patient preference had not impacted on the PAI, as although more than half of patients showed a preference for CAT‐GSH, they would have had better outcomes from CBT‐GSH according to this model.

## DISCUSSION

This paper is the first to test whether better matching of patients to differing GSH could improve the effectiveness of these brief interventions, that had been competently delivered, and where the patients in the main had decided that this was the GSH that they wanted. This study has not been possible in the past due to limited treatment options previously available at step 2 and the study is the first PAI study that has dealt with the issue of patient preference and GSH. The study shows the importance of treatment plurality at step 2 and provides preliminary evidence that it may be possible to better match patients to different GSH treatments. The present study therefore produces an argument for evidence‐based treatment allocation championing patient preference. This is clearly a challenge to a vast array of health policy which states that supporting patient choice regarding treatment is a key part of any quality service offer (Greener, 2009).

### Main findings

Results suggest that within the full sample and subgroup analyses those patients who had their optimal GSH were significantly more likely to have better anxiety outcomes, compared to patients who had their non‐optimal GSH. However, having an optimal treatment did not significantly affect the trajectory of anxiety outcome scores. This difference in results between the full sample and subgroup may be due to the stronger optimal treatment recommendations indicated by the largest predicted benefit (PAI ≥ mean + 1SD). These results suggest there is a subgroup of patients (18%) who have a marked and differential response to GSH and who could benefit markedly from treatment matching. This fits with existing evidence of the use of the PAI in estimating optimal treatments for traditional psychotherapies for depression. DeRubeis et al. (2014) found that patients matched to their optimal treatment (i.e., medication vs. CBT) had superior outcomes and Huibers et al. (2015) mirrored this, but when comparing cognitive therapy with interpersonal psychotherapy. It is recommended that the PAI should continue to be researched within clinical populations to develop robust algorithms for matching patients to their optimal treatments.

Patients who received their indicated non‐optimal treatment had higher average baseline GAD‐7 scores. It is unclear why this is the case; it may be due to the decision to force entry baseline GAD‐7 scores into the PAI model, rather than including them during the variable selection process of the prediction models. Consideration of how GAD‐7 scores may interact with other variables during variable selection should be considered in future studies. The high rate of randomization refusal in the patient preference trial meant there was a lack of randomization in the sample and these differences may also reflect selection biases in the data that were not controlled for.

### Prediction model

Only two variables were prognostic and were both related to baseline clinical severity (i.e., baseline PHQ‐9 score and baseline BAI score). Similar findings have been seen for low‐intensity CBT treatments for anxiety with baseline depression and anxiety identified as significant predictors of outcome (González‐Robles et al., 2021; Lawn et al., 2019; Mathiasen et al., 2018). The remaining variables were prescriptive. Patients who were from a minoritized background, were employed and had a lower socioeconomic status were predicted to have poorer outcomes after CBT‐GSH. Patients who had higher baseline WSAS scores, no self‐reported long‐term condition, were not in the perinatal period, identified as heterosexual and had previously engaged in CBT were predicted to have poorer outcomes after CAT‐GSH.

Some of the prescriptive variables fit with existing evidence, as El Alaoui et al. (2015); Delgadillo et al. (2016) and Mathiasen et al. (2018) all suggest that employment status significantly predicts outcomes. Monthly income has previously been associated with outcomes, but not significantly (Chen et al., 2020). Delgadillo et al. (2017) reported that an accumulation of contextual disadvantages, such as minority ethnic status, may impact negatively on the potential for psychological change and improvement. Furthermore, Delgadillo and Gonzalez Salas Duhne (2020) also found that minoritized ethnic groups had poorer outcomes after CBT. Overall, the variables selected in the model predicting outcomes after CBT‐GSH fit with the wider literature.

Due to the recent development of CAT‐GSH, there was no extant evidence on predictors of outcome. There is also a limited evidence base investigating predictors of outcome after high‐intensity CAT. It is unclear therefore why the variables that were chosen for the CAT‐GSH prediction model were deemed important. On closer review, perinatal status, sexual orientation and previous CAT were significantly skewed. Only 6.2% of the sample were within the perinatal period and 10% identified as not heterosexual. Only three patients (1.4%) had engaged in traditional CAT previously, all of whom chose CAT‐GSH. These may be adding noise rather than useful predictive power and highlight the need to externally validate these models in a new sample before firm conclusions can be drawn or applied in clinical practice.

### Patient preference

Patient preference was not identified as an indicator of outcome. However, the patient preference sample was skewed, with only 6.2% of the sample randomized. Significantly more patients chose CAT‐GSH than CBT‐GSH and influencers of patient treatment preferences do need to be better understood (Kawathekar, 2023). This highlights further questions about the usefulness of treatment allocation via patient preference versus treatment optimization using artificial intelligence (AI). Previous studies suggest that using AI to match patients to treatment has the potential to improve outcomes (Delgadillo & Gonzalez Salas Duhne, 2020). The evidence for the efficacy of patient preference is mixed. While some studies have found no significant effect of patient preference on outcome (e.g., Dunlop et al., 2017), a meta‐analysis found a small significant effect (Swift & Callahan, 2009). Leveraging patient choice enhances motivation to engage in treatment and enhances the therapeutic alliance (Gelhorn et al., 2011).

Optimization through AI could be viewed as currently, the most objective method of matching patients to treatment. Machine learning is becoming more widely used as a method of treatment matching and some studies have suggested that this should be combined with patient preference to ensure a shared and equal decision‐making process (Hamilton et al., 2024). It may be that this is the most cautious and ethical way forward in integrating AI into mental health treatment. To offer treatment based on evidence and then when the patient knows what that treatment entails, then allocate according to that decision. This may be the best‐case scenario for patients with a large PAI for example.

### Practical implications

The GSH model is based on a collaborative approach (Ruth & Spiers, 2023) and clearly the PAI does not take a collaborative approach, but rather uses data to create a decision. Therefore, therapists' and practitioners' attitudes towards integrating treatment selection methods into their everyday clinical practice need to be better understood (Beutler et al., 2016) and also training offered on how to integrate treatment selection into collaborative useful dialogue with patients during the assessment phase. This should adopt an ‘and/both’ approach (i.e., treatment selection going hand in hand with patient preferences) and not an ‘either/or’ approach (i.e., either all treatment selection or all patient preference). Friedl et al. (2020) offer some pointers in terms of the consideration of how the patient's quality of life and functioning needs be considered during decision making, as poor functioning would indicate the need for more intense, face‐to‐face and longer treatment contacts. Service audit would need to capture the rate at which patient preferences are matched to treatment selection decisions and when not. In the original patient preference trial these results are based upon, there was a diligent effort to create effective patient‐centred psychoeducation detailing the style and approach of each GSH, so that patients could make an informed choice. The patient information sheet regarding treatment options went through five iterations and had expert and patient feedback (Kellett et al., 2023). Therefore, the way a treatment selection decision is made needs to be clearly set out for patients in ways that are easily understood, and very clear descriptions of the indicated treatments created to enable shared decision making (Ruth & Spiers, 2023). Otherwise, the risk is that patients feel ‘done to.’ Hamilton et al.'s (2024) example in coronary care highlights the need for patient involvement in the design of the treatment selection feedback tool.

### Limitations

Significantly more patients received CAT‐GSH than CBT‐GSH and so external cross validation of the prediction model was unfeasible. A decision was made to maximize the sample size for training the models. This algorithm should therefore be viewed as a ‘proof of concept’ as the sample sizes were suboptimal and underpowered (Fransén et al., 2022; Lutz et al., 2018). While internal cross validation increased the reliability of the predictive model, external cross validation in an independent sample would be required before clinical utility can be determined.

### Treatment options and future research

These findings suggest some patients could have had better outcomes if they had engaged in a different version of GSH. This is an important finding as currently in NHS services, CBT‐GSH is the only treatment widely available at step 2. Offering treatment choice to evidence‐based GSH needs to be backed up with clear psychoeducational materials, so that patient preferences are scaffolded by the clearest descriptions and the best evidence. The algorithm developed within this study for matching patients to each GSH should be evaluated with larger samples. Use of routine outcome data from NHS Talking Therapies services implementing the CAT‐GSH intervention alongside standard CBT‐GSH could be used to validate these models in a larger sample using established statistical matching methods (e.g., propensity score matching) to better control for baseline differences.

## CONCLUSION

Results from this study suggest that matching patients to treatments can improve outcomes for brief GSH, particularly for a subsample of patients with large PAI scores. Lorimer et al. (2024) highlighted the ‘revolving door’ of IAPT and that 13.7% of patients returned to the service within 1–5 years. Better treatment matching improves outcomes and therefore this could have an impact on the treatment return rate and possibly create more efficient services. This study supports the use of machine learning and prediction models to enable treatment matching, suggesting that using trained models holds potential. When there is no strongly recommended treatment, patient preference should guide treatment allocation. The study again underlines the need for treatment plurality at Step 2 of the NHS Talking Therapies. Effective matching of patients to treatments at step 2 is particularly important due to their ubiquity and their brevity, as there is less time and opportunity to adapt the GSH to the person. Further research is clearly indicated with larger and more balanced samples to identify who GSH works best for.

## AUTHOR CONTRIBUTIONS

**Caroline Wojnarowski:** Writing – original draft; writing – review and editing; formal analysis; project administration; data curation. **Melanie Simmonds‐Buckley:** Conceptualization; writing – original draft; writing – review and editing; supervision; formal analysis; project administration; methodology. **Stephen Kellett:** Funding acquisition; writing – original draft; methodology; writing – review and editing; supervision; project administration.

## FUNDING INFORMATION

The original clinical trial this current study was based on was funded by the Association of Cognitive Analytic Therapists (ACAT) and Catalyse Ltd.

## CONFLICT OF INTEREST STATEMENT

The authors declare no conflicts of interest.

## Supporting information


Data S1.

## Data Availability

The data and the analysis are available from the corresponding author on request.

## References

[bjc12508-bib-0001] Beck, A. T. , & Steer, R. A. (1993). Beck anxiety inventory manual. Psychological Corporation.

[bjc12508-bib-0002] Bennett‐Levy, J. , Richards, D. A. , & Farrand, P. (2010). Low intensity CBT interventions: A revolution in mental health care. In J. Bennett‐Levy & P. Farrand (Eds.), Oxford guide to low intensity CBT interventions (Vol. 1, pp. 3–18). Oxford University.

[bjc12508-bib-0003] Beutler, L. E. , Someah, K. , Kimpara, S. , & Miller, K. (2016). Selecting the most appropriate treatment for each patient. International Journal of Clinical and Health Psychology, 16(1), 99–108. 10.1016/j.ijchp.2015.08.001 30487854 PMC6225028

[bjc12508-bib-0004] Blanca, M. J. , Alarcón Postigo, R. , Arnau Gras, J. , Bono Cabré, R. , & Bendayan, R. (2017). Non‐normal data: Is ANOVA still a valid option? Psicothema, 29(4), 552–557. 10.7334/psicothema2016.383 29048317

[bjc12508-bib-0005] Boogar, I. R. , Rezaei, A. M. , & Yosefi, A. (2013). The effectiveness of cognitive analytic therapy on the severity of symptoms in patients with obsessive‐compulsive disorder. Practice in Clinical Psychology, 1, 197–204.

[bjc12508-bib-0007] Breiman, L. (2001). Random forests. Machine Learning, 45(1), 5–32. 10.1023/A:1010933404324

[bjc12508-bib-0008] Chan, S. W. Y. , & Adams, M. (2014). Service use, dropout rate and clinical outcomes: A comparison between high and low intensity treatments in an IAPT service. Cognitive and Behavioural Psychotherapy, 42(6), 747–759. 10.1017/S1352465813000544 24382088

[bjc12508-bib-0009] Chen, H. , Rodriguez, M. A. , Qian, M. , Kishimoto, T. , Lin, M. , & Berger, T. (2020). Predictors of treatment outcomes and adherence in internet‐based cognitive behavioral therapy for social anxiety in China. Behavioural and Cognitive Psychotherapy, 48(3), 291–303. 10.1017/S1352465819000730 31928568

[bjc12508-bib-0011] Collins, G. S. , Reitsma, J. B. , Altman, D. G. , & Moons, K. G. (2015). Transparent reporting of a multivariable prediction model for individual prognosis or diagnosis (TRIPOD): The TRIPOD statement. Journal of British Surgery, 102(3), 148–158. 10.1111/1471-0528.13244 25627261

[bjc12508-bib-0013] Coull, G. , & Morris, P. G. (2011). The clinical effectiveness of CBT‐based guided self‐help interventions for anxiety and depressive disorders: A systematic review. Psychological Medicine, 41(11), 2239–2252. 10.1017/S0033291711000900 21672297

[bjc12508-bib-0014] Cuijpers, P. , Donker, T. , Van Straten, A. , Li, J. , & Andersson, G. (2010). Is guided self‐help as effective as face‐to‐face psychotherapy for depression and anxiety disorders? A systematic review and meta‐analysis of comparative outcome studies. Psychological Medicine, 40, 1943–1957. 10.1017/S0033291710000772 20406528

[bjc12508-bib-0015] Damen, J. A. , Hooft, L. , Schuit, E. , Debray, T. P. , Collins, G. S. , Tzoulaki, I. , & Moons, K. G. (2016). Prediction models for cardiovascular disease risk in the general population: Systematic review. British Medical Journal, 353, i2416. 10.1136/bmj.i2416 27184143 PMC4868251

[bjc12508-bib-0016] Deisenhofer, A. K. , Delgadillo, J. , Rubel, J. A. , Böhnke, J. R. , Zimmermann, D. , Schwartz, B. , & Lutz, W. (2018). Individual treatment selection for patients with posttraumatic stress disorder. Depression and Anxiety, 35(6), 541–550. 10.1002/da.22755 29659106

[bjc12508-bib-0017] Delgadillo, J. , & Gonzalez Salas Duhne, P. (2020). Targeted prescription of cognitive–behavioral therapy versus person‐centred counselling for depression using a machine learning approach. Journal of Consulting and Clinical Psychology, 88(1), 14–24. 10.1037/ccp0000476 31841021

[bjc12508-bib-0018] Delgadillo, J. , Huey, D. , Bennett, H. , & McMillan, D. (2017). Case complexity as a guide for psychological treatment selection. Journal of Consulting and Clinical Psychology, 85(9), 835–853. 10.1037/ccp0000231 28857592

[bjc12508-bib-0019] Delgadillo, J. , & Lutz, W. (2020). A development pathway towards precision mental health care. JAMA Psychiatry, 77(9), 889–890. 10.1001/jamapsychiatry.2020.1048 32459326

[bjc12508-bib-0020] Delgadillo, J. , Moreea, O. , & Lutz, W. (2016). Different people respond differently to therapy: A demonstration using patient profiling and risk stratification. Behaviour Research and Therapy, 79, 15–22. 10.1016/j.brat.2016.02.003 26937855

[bjc12508-bib-0021] Delgadillo, J. , Rhodes, L. , Moreea, O. , McMillan, D. , Gilbody, S. , Leach, C. , Lucock, M. , Lutz, W. , & Ali, S. (2018). Relapse and recurrence of common mental health problems after low intensity cognitive behavioural therapy: The WYLOW longitudinal cohort study. Psychotherapy and Psychosomatics, 87(2), 116–117. 10.1159/000485386 29462816

[bjc12508-bib-0022] DeRubeis, R. J. , Cohen, Z. D. , Forand, N. R. , Fournier, J. C. , Gelfand, L. A. , & Lorenzo‐Luaces, L. (2014). The personalized advantage index: Translating research on prediction into individualized treatment recommendations. A demonstration. PLoS One, 9(1), e83875. 10.1371/journal.pone.0083875 24416178 PMC3885521

[bjc12508-bib-0023] Dunlop, B. W. , Kelley, M. E. , Aponte‐Rivera, V. , Mletzko‐Crowe, T. , Kinkead, B. , Ritchie, J. C. , Nemeroff, C. B. , Craighead, W. E. , & Mayberg, H. S. (2017). Effects of patient preferences on outcomes in the predictors of remission in depression to individual and combined treatments (PReDICT) study. American Journal of Psychiatry, 174(6), 546–556. 10.1176/appi.ajp.2016.16050517 28335624 PMC6690210

[bjc12508-bib-0024] Efron, B. (1982). The jackknife, the bootstrap and other resampling plans. Society for Industrial and Applied Mathematics (SIAM).

[bjc12508-bib-0025] El Alaoui, S. E. , Ljótsson, B. , Hedman, E. , Kaldo, V. , Andersson, E. , Rück, C. , Andersson, G. , & Lindefors, N. (2015). Predictors of symptomatic change and adherence in internet‐based cognitive behaviour therapy for social anxiety disorder in routine psychiatric care. PLoS One, 10(4), e0124258. 10.1371/journal.pone.0124258 25893687 PMC4404057

[bjc12508-bib-0026] Fransén, J. , Lundin, J. , Fredén, F. , & Huss, F. (2022). A proof‐of‐concept study on mortality prediction with machine learning algorithms using burn intensive care data. Scars, Burns and Healing, 8, 20595131211066585. 10.1177/20595131211066585 PMC8859689 35198237

[bjc12508-bib-0027] Friedl, N. , Krieger, T. , Chevreul, K. , Hazo, J. B. , Holtzmann, J. , Hoogendoorn, M. , Kleiboer, A. , Mathiasen, K. , Urech, A. , Riper, H. , & Berger, T. (2020). Using the personalized advantage index for individual treatment allocation to blended treatment or treatment as usual for depression in secondary care. Journal of Clinical Medicine, 9(2), 490. 10.3390/jcm9020490 32054084 PMC7073663

[bjc12508-bib-0029] Gelhorn, H. L. , Sexton, C. C. , & Classi, P. M. (2011). Patient preferences for treatment of major depressive disorder and the impact on health outcomes: A systematic review. Primary Care Companion for CNS Disorders, 13(5), PCC.11r01161. 10.4088/PCC.11r01161 PMC3267514 22295273

[bjc12508-bib-0030] González‐Robles, A. , Suso‐Ribera, C. , Díaz‐García, A. , García‐Palacios, A. , Castilla, D. , & Botella, C. (2021). Predicting response to transdiagnostic iCBT for emotional disorders from patient and therapist involvement. Internet Interventions, 25, 100420. 10.1016/j.invent.2021.100420 34401379 PMC8350608

[bjc12508-bib-0031] Greener, I. (2009). Towards a history of choice in UK health policy. Sociology of Health & Illness, 31(3), 309–324. 19055589 10.1111/j.1467-9566.2008.01135.x

[bjc12508-bib-0032] Hamilton, D. E. , Albright, J. , Seth, M. , Painter, I. , Maynard, C. , Hira, R. S. , Sukul, D. , & Gurm, H. S. (2024). Merging machine learning and patient preference: A novel tool for risk prediction of percutaneous coronary interventions. European Heart Journal, 45, 601–609. 10.1093/eurheartj/ehad836 38233027

[bjc12508-bib-0033] Headley, E. , Kellett, S. , Bee, C. , Lancashire, J. , Aadahl, V. , Bone, C. , & Power, N. (2024). Types and mechanisms of idiographic change during guided self‐help for anxiety. Psychology and Psychotherapy, 97, 498–517. 38924285 10.1111/papt.12536

[bjc12508-bib-0034] Huibers, M. J. , Cohen, Z. D. , Lemmens, L. H. , Arntz, A. , Peeters, F. P. , Cuijpers, P. , & DeRubeis, R. J. (2015). Predicting optimal outcomes in cognitive therapy or interpersonal psychotherapy for depressed individuals using the personalized advantage index approach. PLoS One, 10(11), e0140771. 10.1371/journal.pone.0140771 26554707 PMC4640504

[bjc12508-bib-0036] Jacobson, N. S. , Roberts, L. J. , Berns, S. B. , & McGlinchey, J. B. (1999). Methods for defining and determining the clinical significance of treatment effects: Description, application, and alternatives. Journal of Consulting and Clinical Psychology, 67(3), 300–307. 10.1037/0022-006X.67.3.300 10369050

[bjc12508-bib-0037] Kawathekar, U. S. (2023). From expertise to collaboration: Understanding the role of patient preferences in clinical decision making . JOSPT. https://www.jospt.org/do/10.2519/jospt.blog.20230726/full/#:~:text=Role%20Preferences%20in%20Clinical%20Decision,relationship%20with%20the%20healthcare%20provider

[bjc12508-bib-0038] Kellett, S. , Bee, C. , Aadahl, V. , Headley, E. , & Degadillo, J. (2021). A pragmatic patient preference trial of cognitive behavioural versus cognitive analytic guided self‐help for anxiety disorders. Behavioural and Cognitive Psychotherapy, 49, 104–111.

[bjc12508-bib-0039] Kellett, S. , Bee, C. , Smithies, J. , Aadahl, V. , Simmonds‐Buckley, M. , Power, N. , Duggan‐Williams, C. , Fallon, N. , & Delgadillo, J. (2023). Cognitive–behavioural versus cognitive–analytic guided self‐help for mild‐to‐moderate anxiety: A pragmatic, randomised patient preference trial. British Journal of Psychiatry, 223(3), 438–445. 10.1192/bjp.2023.78 PMC10895510 37395600

[bjc12508-bib-0040] Kellett, S. , Simmonds‐Buckley, M. , Limon, E. , Hague, J. , Hughes, L. , Stride, C. , & Millings, A. (2021). Defining the assessment and treatment competencies to deliver low‐intensity cognitive behavior therapy: A multi‐centre validation study. Behavior Therapy, 52(1), 15–27. 10.1016/j.beth.2020.01.006 33483113

[bjc12508-bib-0041] Kroenke, K. , Spitzer, R. L. , Williams, J. B. , Monahan, P. O. , & Löwe, B. (2007). Anxiety disorders in primary care: Prevalence, impairment, comorbidity, and detection. Annals of Internal Medicine, 146(5), 317–325. 17339617 10.7326/0003-4819-146-5-200703060-00004

[bjc12508-bib-0042] Kroenke, K. , Spitzer, R. L. , & Williams, J. B. W. (2001). The PHQ‐9: Validity of a brief depression measure. Journal of General Internal Medicine, 16, 606–613. 11556941 10.1046/j.1525-1497.2001.016009606.x PMC1495268

[bjc12508-bib-0043] Kuhn, M. (2008). Building predictive models in R using the caret package. Journal of Statistical Software, 28(5), 1–26. 10.18637/jss.v028.i05 27774042

[bjc12508-bib-0044] Kursa, M. B. , & Rudnicki, W. R. (2010). Feature selection with the Boruta package. Journal of Statistical Software, 36(11), 1–13. 10.18637/jss.v036.i11

[bjc12508-bib-0045] Lawn, S. , Huang, N. , Zabeen, S. , Smith, D. , Battersby, M. , Redpath, P. , Glover, F. , Venning, A. , Cameron, J. , & Fairweather‐Schmidt, K. (2019). Outcomes of telephone‐delivered low‐intensity cognitive behaviour therapy (LiCBT) to community dwelling Australians with a recent hospital admission due to depression or anxiety: MindStep. BMC Psychiatry, 19(1), 2. 10.1186/s12888-018-1987-1 30606169 PMC6319009

[bjc12508-bib-0046] Lewis, C. , Pearce, J. , & Bisson, J. I. (2012). Efficacy, cost‐effectiveness and acceptability of self‐help interventions for anxiety disorders: Systematic review. British Journal of Psychiatry, 200(1), 15–21. 10.1192/bjp.bp.110.084756 22215865

[bjc12508-bib-0047] Lorimer, B. , Kellett, S. , Giesemann, J. , Lutz, W. , & Delgadillo, J. (2024). An investigation of treatment return after psychological therapy for depression and anxiety. Behavioural and Cognitive Psychotherapy, 52(2), 149–162. 10.1017/S1352465823000322 37563726

[bjc12508-bib-0048] Lutz, W. , Schwartz, B. , Hofmann, S. G. , Fisher, A. J. , Husen, K. , & Rubel, J. A. (2018). Using network analysis for the prediction of treatment dropout in patients with mood and anxiety disorders: A methodological proof‐of‐concept study. Scientific Reports, 8(1), 7819. 10.1038/s41598-018-25953-0 29777110 PMC5959887

[bjc12508-bib-0050] Mathiasen, K. , Riper, H. , Andersen, T. E. , & Roessler, K. K. (2018). Guided internet‐based cognitive behavioral therapy for adult depression and anxiety in routine secondary care: Observational study. Journal of Medical Internet Research, 20(11), e10927. 10.2196/10927 30487118 PMC6291683

[bjc12508-bib-0051] Meadows, J. , & Kellett, S. (2017). Development and evaluation of cognitive analytic guided self‐help (CAT‐SH) for use in IAPT services. Behavioural and Cognitive Psychotherapy, 45(3), 266–284. 10.1017/S1352465816000485 28325165

[bjc12508-bib-0052] Miles, J. , & Shelvin, M. (2001). Applying regression. Sage Publications.

[bjc12508-bib-0053] Milosevic, I. , Levy, H. C. , Alcolado, G. M. , & Radomsky, A. S. (2015). The treatment acceptability/adherence scale: Moving beyond the assessment of treatment effectiveness. Cognitive Behaviour Therapist, 44, 456–469. 10.1080/16506073.2015.1053407 26091250

[bjc12508-bib-0054] Moggia, D. , Saxon, D. , Lutz, W. , Hardy, G. E. , & Barkham, M. (2023). Applying precision methods to treatment selection for moderate/severe depression in person‐centered experiential therapy or cognitive behavioral therapy. Psychotherapy Research, 1–16. 10.1080/10503307.2023.2269297. Epub ahead of print. 37917065

[bjc12508-bib-0055] Mundt, J. C. , Marks, I. M. , Shear, M. K. , & Greist, J. M. (2002). The work and social adjustment scale: A simple measure of impairment in functioning. British Journal of Psychiatry, 180(5), 461–464. 10.1192/bjp.180.5.461 11983645

[bjc12508-bib-0056] National Collaborating Centre for Mental Health . (2018). The improving access to psychological therapies manual . https://www.rcpsych.ac.uk/docs/default‐source/improving‐care/nccmh/iapt/the‐iapt‐manual‐v6.pdf?sfvrsn=79ece7e5_2

[bjc12508-bib-0057] National Institute for Health and Clinical Excellence (NICE) . (2014). Cardiovascular disease: Risk assessment and reduction, including lipid modification. National Institute for Health and Care Excellence.

[bjc12508-bib-0058] National Institute of Health and Clinical Excellence (NICE) . (2009). Clinical guideline 90. Depression: The Treatment and Management of Depression in Adults . http://www.nice.org.uk/nicemedia/pdf/CG90NICEguideline.pdf 31886986

[bjc12508-bib-0059] National Institute of Health and Clinical Excellence (NICE) . (2011). Common mental health disorders: Identification and pathways to care. Clinical guideline 123. National Institute for Health and Clinical Excellence. 31877005

[bjc12508-bib-0060] Priemer, M. , & Talbot, F. (2013). CBT guided self‐help compares favourably to gold standard therapist‐administered CBT and shows unique benefits over traditional treatment. Behaviour Change, 30(4), 227–240. 10.1017/bec.2013.22

[bjc12508-bib-0061] R Core Team . (2021). *R: A language and environment for statistical computing* [computer software]. R foundation for Statistical Computing. https://www.R‐project.org/

[bjc12508-bib-0062] Richards, D. , & Whyte, M. (2011). Reachout. National programme student materials to support the delivery of training for psychological wellbeing practitioners delivering low intensity interventions. Rethink Publishing.

[bjc12508-bib-0065] RStudio Team . (2021). *Rstudio: Integrated development for R* [computer software]. RStudio, Inc. http://www.rstudio.com/

[bjc12508-bib-0066] Ruth, E. , & Spiers, J. (2023). A pragmatic guide to low intensity psychological therapy. Elsevier Publishing.

[bjc12508-bib-0069] Schwartz, B. , Cohen, Z. D. , Rubel, J. A. , Zimmermann, D. , Wittmann, W. W. , & Lutz, W. (2021). Personalized treatment selection in routine care: Integrating machine learning and statistical algorithms to recommend cognitive behavioral or psychodynamic therapy. Psychotherapy Research, 31(1), 33–51. 10.1080/10503307.2020.1769219 32463342

[bjc12508-bib-0072] Stekhoven, D. J. , & Bühlmann, P. (2012). MissForest—Non‐parametric missing value imputation for mixed‐type data. Bioinformatics, 28(1), 112–118. 10.1093/bioinformatics/btr597 22039212

[bjc12508-bib-0073] Swift, J. K. , & Callahan, J. L. (2009). The impact of client treatment preferences on outcome: A meta‐analysis. Journal of Clinical Psychology, 65(4), 368–381. 10.1002/jclp.20553 19226606

[bjc12508-bib-0074] Tang, C. , Garreau, D. , & von Luxburg, U. (2018). When do random forests fail? Advances in Neural Information Processing Systems, 31, 1–11.

[bjc12508-bib-0075] Torgerson, D. , & Sibbald, B. (1998). Understanding controlled trials: What is a patient preference trial? British Medical Journal, 316, 360–365. 10.1136/bmj.316.7128.360 9487173 PMC2665528

[bjc12508-bib-0076] Turpin, G. (2010). Good practice guidelines on the use of self‐help materials within IAPT services . IAPT Document.

[bjc12508-bib-0077] Tzouramanis, P. , Adamopoulou, A. , Bozikas, V. , Voikli, M. , Zagora, C. , Lombtzianidou, M. , Mamouzelos, E. , & Garyfallos, G. (2010). Evaluation of cognitive‐analytic therapy (CAT) outcome in patients with panic disorder. Psychiatrike, 21, 287–293. 21914611

[bjc12508-bib-0078] University College London . (2014). PWP best practice guide . https://www.ucl.ac.uk/pals/sites/pals/files/pwp_training_review_appendix_8_‐_pwp_best_practice_guide.pdf

[bjc12508-bib-0080] Wray, A. , Kellett, S. , Bee, C. , Smithies, J. , Aadahl, V. , Simmonds‐Buckley, M. , & McElhatton, C. (2022). The acceptability of cognitive analytic guided self‐help in an improving access to psychological therapies service. Behavioural and Cognitive Psychotherapy, 50(5), 493–507. 10.1017/S1352465822000194 35575218

[bjc12508-bib-0081] Zou, H. , & Hastie, T. (2005). Regularization and variable selection via the elastic net. Journal of the Royal Statistical Society, Series B: Statistical Methodology, 67(2), 301–320. 10.1111/j.1467-9868.2005.00503.x

