# Supplementary material for: Predicting optimal treatment allocation for cognitive analytic‐guided self‐help versus cognitive behavioural‐guided self‐help
Source: Br J Clin Psychol. 2024 Oct 23;64(2):355–70. doi: 10.1111/bjc.12508 (PMC12057329; doi:10.1111/bjc.12508)
Supplement: Supplementary file 1 — Data S1. [file BJC-64-355-s001.docx]

**Appendix S1**. Inclusion/Exclusion Criteria from Kellett et al., (2023)

| Inclusion | Exclusion |
| --- | --- |
| Self-referred or been referred by their General Practitioner/other health or social care professional for the treatment of a common mental health problem. | Currently taking part in another NHS Talking Therapies step 2 intervention. |
| Met criteria for an anxiety disorder based on the Mini International Neuropsychiatric Interview (MINI; Sheehan et al., 1998). | Didn’t meet the criteria for an anxiety disorder or “caseness” on the BAI. |
| Scored above the cut-off for clinically significant symptoms on Beck’s Anxiety Inventory (BAI; Beck & Steer, 1993). | Met criteria for comorbid depression and anxiety disorder, where the depression is the main concern. |
| Want to engage in GSH for anxiety. | Had a severe/chronic mental health difficulty - engaged with secondary mental health care services or Diagnosis of social phobia/post-traumatic stress disorder (treated at step 3). |
| Motivated to engage in treatment and able to attend six face-to-face GSH sessions. | Unable to read and write or need an interpreter. |

**Appendix S2**. Data collected within initial Kellett et al., (2023) trial and suitable for analysis in the current study.

| Variable | Method of Measurement (Transformed) |
| --- | --- |
| Age | Continuous |
| Ethnicity | White British or Minoritised Ethnicity |
| Employment Status | In Employment or Not Employed |
| Sexual Orientation | Heterosexual or Not Heterosexual |
| Gender | Male or Female |
| Identification of Long-Term Condition (LTC) | Self-reported LTC or no LTC |
| Veteran Status | Veteran or Not a Veteran |
| Perinatal Status | Pregnant or Not Pregnant |
| Psychotropic Medication | Not Taking Medication or Taking Medication |
| Indices of Multiple Deprivation (IMD) | Continuous |
| GAD-7 baseline severity | Continuous |
| PHQ-9 baseline severity | Continuous |
| WSAS baseline severity | Continuous |
| BAI baseline severity | Continuous |
| Previous Treatment | Previous Treatment Disclosed or No Previous Treatment |
| Previous CAT | Had CAT or Not Had CAT |
| Previous CBT | Had CBT or Not Had CBT |
| Allocation Choice | Treatment Preference or Randomisation |

**APPENDIX S3** The measures and their properties used in the Kellett at al., (2023) trial

**Beck Anxiety Inventory** (BAI) is a tool to assess symptoms of anxiety. Items were specifically selected to separate anxiety from depression. It has 21 items with a suggested cut-off for clinically significant anxiety as 16 (Beck & Steer, 1993). The BAI has high internal consistency and has good reliability (Fydrich, Dowdall, & Chambless, 1992).

**Generalised Anxiety Disorder 7** (GAD-7) is an assessment tool to support the diagnosis of generalised anxiety disorder. It has 7 items with a maximum score of 21. Scores of 5, 10, and 15 are taken as the cut-off points for mild, moderate and severe anxiety, respectively. Using the threshold score of 10, the GAD-7 has a sensitivity of 89% and a specificity of 82% for GAD (Kroenke et al., 2007). Spitzer et al. (2006), found the questionnaire has good validity and reliability. It is routinely used within NHS Talking Therapies.

**Patient Health Questionnaire** **9** (PHQ- 9) is an assessment tool to support the recognition of depression in patients. It has 9 items with a maximum score of 27. Total scores of 5, 10, 15, and 20 represent cut-offs for mild, moderate, moderately severe and severe depression, respectively. It can be repeated over time to monitor changes and has been assessed as having excellent test-retest reliability (Kroenke et al., 2001).

**Work and Social Adjustment Scale** (WSAS) is a 5-item self-report measure that provides the impact of a disorder on daily life, it has a maximum score of 40. Scores between 10 and 20 are associated with significant functional impairment but less severe clinical symptomatology, over 20 suggests moderately severe psychopathology. Mundt et al. (2002) reported that the WSAS is reliable, valid and sensitive to change.

**Appendix S4.** *STROBE diagram.*

**Appendix S5.** Sample size calculation

The sample size calculation proposed by Riley et al., (2019) provides an equation for multi-variate prediction models of continuous outcomes based on the required number of predictors, expected explained variance (R^2^) and allowing for up to 10% out-of-sample prediction shrinkage. A variable selection procedure was applied prior to developing the models and it was anticipated that approximately seven of the predictors would be retained. Literature on previous psychological treatment prediction models for continuous outcomes reported R^2^ values in the range of .35 to .45, indicating that baseline characteristics tend to explain on average 40% of variance in post-treatment scores (Friedl et al., 2020; Salomonsson et al., 2020; Schwartz et al., 2021; Senger et al., 2022). On this basis, a sample size calculation for a predictive model using an estimate value of R^2^ =0.40, including 7 predictors and allowing for up to 10% shrinkage would require 79 patients in each treatment group.

**Appendix S6.** Supplementary Data Analysis Information

Random Forest (Boruta method)

Random forest builds multiple decision trees using a bagging method (a combination variable and bootstrapping samples; Breiman, 2001). A variable is selected to create a node, this is then analysed to establish threshold values to split observations (a tree branch). A new variable is then selected for each branch with remaining observations split through recursive partitioning. Various decision trees are build based on bootstrapped datasets and a random selection of variables. Data not included in the bootstrapped datasets are the out-of-the-bag (OOB) cases. This prevents overfitting of the model to the current dataset. The use of random selection reduces the overriding effects of stronger predictive variables as it uses information from weaker predictors. The importance of each predictor is expressed as a “mean decrease in accuracy” which presents how much a variable increases or decreases the models accuracy. Such approaches handle multicollinearity well and can be well suited to multivariable prediction models.

One of the limitations of standard random forest variable selection is that it provides a rank-ordered list of variable importance but does not remove any variables. Researchers are required to set criteria for what threshold will be used to retain predictors in the model and this has been applied in different ways, including retaining only predictors reaching 90% importance in the model (Schwartz et al. 2021) or positive scores of mean decrease in prediction accuracy (Moggia et al. 2023). The Boruta method aims to address this issue by extending the random forest approach to include an embedded variable selection criterion. Shadow variables (one continuous and one categorical) are created based on the distributions of other variables in the dataset and included in the model as a ‘noise’ variable (i.e., have no actual predictive power). Only variables which are ranked higher than one (tentative inclusion) or both (confirmed inclusion) shadow variables are deemed to have reliable predictive power over and above noise and are retained.

Elastic Net Regularisation

This method combines both the Least Absolute Shrinkage and Selection Operator (LASSO) and Ridge penalties. LASSO penalisation shrinks coefficients with no predictive value to zero and Ridge penalisation shrinks coefficients with less predictive value towards zero, therefore not excluding any predictors. Elastic Net reduces overfitting of the model, increases prediction accuracy and model generalisability (Zou & Hastie, 2005). Multiple potential models were tested using 10-fold cross-validation (Rodriguez et al., 2009), this enables selection of predictors to be less influenced by extreme outliers (Breckler, 1990; MacCallum et al., 1992). The Elastic net model which had the best accuracy and lowest prediction error (i.e., best combination of LASSO and Ridge penalties was chosen). The best fitting model was based on the one standard error rule, the most parsimonious model whose error was no more than one standard error above the error of the best model was chosen. As a result, this chosen model was more likely to generalise to other data and not be too specific (overfitted) to the current data, which might have been the case if the “best model” was chosen.

**Appendix S7.** ANOVA assumption testing results

Test of Normality Full Sample (*significance at p<0.05)

| Time | Group | Shapiro-Wilk Statistic | df | Significance |
| --- | --- | --- | --- | --- |
| Baseline | *Optimal* | .970 | 131 | .006* |
|  | *Non-optimal* | .941 | 78 | .001* |
| Post-treatment | *Optimal* | .955 | 131 | <.001* |
|  | *Non-optimal* | .957 | 78 | .010* |
| Follow-up | *Optimal* | .949 | 131 | <.001* |
|  | *Non-optimal* | .967 | 78 | .041* |

Test of Normality Subgroup (*significance at p<0.05)

| Time | Group | Shapiro-Wilk Statistic | df | Significance |
| --- | --- | --- | --- | --- |
| Baseline | *Optimal* | .968 | 26 | .575 |
|  | *Non-optimal* | .829 | 11 | .022* |
| Post-treatment | *Optimal* | .877 | 26 | .005* |
|  | *Non-optimal* | .943 | 11 | .554 |
| Follow-up | *Optimal* | .933 | 26 | .092 |
|  | *Non-optimal* | .909 | 11 | .235 |

| Time | Levene Statistic | df | Significance |
| --- | --- | --- | --- |
| *Baseline* | .521 | 1, 207 | .471 |
| *Post-treatment* | .359 | 1, 207 | .550 |
| *Follow-up* | .869 | 1, 207 | .352 |

Test of Homogeneity of Variance Full Sample

| Time | Levene Statistic | Df | Significance |
| --- | --- | --- | --- |
| *Baseline* | 1.676 | 1, 35 | .204 |
| *Post-treatment* | .055 | 1, 35 | .816 |
| *Follow-up* | .254 | 1, 35 | .617 |

Test of Homogeneity of Variance Subgroup
